# Protocol for meta‐analysis of temperature reduction in animal models of cardiac arrest

**DOI:** 10.1002/ebm2.14

**Published:** 2016-05-03

**Authors:** H. Olai, G. Thornéus, H. Watson, M.R. Macleod, H. Friberg, J. Rhodes, N. Nielsen, T. Cronberg, T. Deierborg

**Affiliations:** ^1^ Department of Experimental Medical ScienceExperimental Neuroinflammation LaboratoryLundSweden; ^2^ Centre for Clinical Brain SciencesUniversity of EdinburghEdinburghUK; ^3^ Department of Anesthesia and Intensive CareSkåne University HospitalLundSweden; ^4^ Department of Clinical SciencesLund UniversityLundSweden; ^5^ Department of Critical Care, Anaesthesia and Pain MedicineUniversity of EdinburghEdinburghUK; ^6^ Intensive Care and AnaesthesiaWestern General HospitalEdinburghUK; ^7^ Department of Anaesthesia and Intensive CareHelsingborg HospitalHelsingborgSweden; ^8^ Department of NeurologySkåne University HospitalLundSweden

**Keywords:** temperature management, hypothermia, cardiac arrest, global ischaemia, meta‐analysis, animals

## Abstract

Targeted temperature management (TTM) of 32–34 °C has been the standard treatment for out‐of‐hospital cardiac arrest since clinical trials in 2002 showed benefits to survival and neurological outcome. Recently, this treatment has been challenged by another clinical trial showing no difference in outcome between TTM of 33 °C and 36 °C. This protocol describes the methodology for a meta‐analysis detailing temperature‐reducing interventions to treat global ischaemia in animal models. By combining relevant data sets in the literature, we will explore the experimental evidence for TTM. Our aims are to explain possible translational gaps and provide methodological considerations for future experimental research and clinical trials.

## Project Stage at Time of Protocol Submission for Peer‐Reviewed Publication

**Table:** 

Project stage	Started	Completed
Preliminary searches	Yes	Yes
Abstract screening using checklist	Yes	No
Full‐text review of included abstracts	No	No
Data extraction from included papers	No	No
Data analysis	No	No
Manuscript writing	No	No

## Research Question

### background

In Europe, attempted resuscitation in out‐of‐hospital cardiac arrest is estimated to have an incidence close to 40 per 100,000 inhabitants per year.^1^ It is a severe medical condition with high mortality rates, and survivors frequently suffer neurological deficit.^2^ Immediately following the onset of cardiac arrest, that is, global brain ischaemia, energy depletion in the brain tissue leads to a severe failure of cellular homeostasis, with subsequent cell death and inflammation in the brain. Experimental animal models have shown that the body temperature has a large effect on the extent of brain damage that develops following global ischaemia.^3^, ^4^, ^5^, ^6^, ^7^ Hyperthermia is associated with increased damage,^4^^,^ 8 ^,^ 9 whilst hypothermia has a protective effect in several injury models.^3^^,^ 5 ^,^ 7 In 2002, two landmark randomized clinical trials testing the effects of induced hypothermia in patients with cardiac arrest were published.^10^^,^ 11 The studies showed improved neurological function^10^^,^ 11 and increased survival^10^ in patients cooled to 33 °C for 12–24 hours compared to patients with no hypothermia treatment. These studies, together with a Cochrane review,^12^ have had a major impact on the international guidelines. Since 2003, therapeutic hypothermia has been the recommended treatment for patients who regain circulation after cardiac arrest but remain in a coma.^13^ However, a third clinical trial has recently challenged the concept of *therapeutic* hypothermia. The targeted temperature management (TTM) trial was conducted to increase the evidence base for the concept of temperature regulation post‐cardiac arrest and to help determine the optimal target temperature in this patient population. The TTM trial included a large cohort of cardiac arrest patients (n = 939) and found no benefit from hypothermia at 33 °C as compared to controlled temperature at 36 °C for the following parameters: survival,^14^ detailed neurological outcome^15^ or release of neuron‐specific enolase.^16^ In addition, the THAPCA‐OH trial reported that hypothermia at 33 °C after cardiac arrest in children, compared with normothermia at 36.8 °C, did not confer any significant benefit in survival, with good functional outcome at 1 year.^17^ Another meta‐analysis on clinical trials also questions the supporting evidence for induced hypothermia after cardiac arrest, finding that the quality of evidence was low in contrast to previous Cochrane reviews.^18^ Importantly, the European Resuscitation Council (ERC) guidelines published in October 2015 have updated previous recommendations on post‐resuscitation care to include an option for a constant target temperature between 32 °C and 36 °C.^19^ Furthermore, the terms TTM or temperature control rather than therapeutic hypothermia are recommended. To date, there is no *preclinical* meta‐analysis investigating the effects of temperature reduction in relation to global ischaemia. Overall, the authors hope to explain possible translational gaps between animal and patient models, with the aim of informing methodological considerations for future experimental research and clinical trials.

### research question

What are the effects of temperature reduction in animal models of cardiac arrest on neurobehavioural outcome, histological outcome and mortality?

## Objectives

1. To assess the quality of individual studies investigating temperature reduction in animal models of cardiac arrest.
2. To assess the efficacy (reported as histological outcome, neurobehavioural outcome or mortality) of temperature reduction in animal models of cardiac arrest and the impact of study design factors.
3. To assess the range of supporting evidence using a modified Stroke Therapy Academic Industry Roundtable (STAIR) criteria.

With the results from 1–3, the authors aim to explore and discuss possible:

- Translational gaps in the available data on the effect of temperature reduction in animal models of cardiac arrest.
- Sources of bias and sources of heterogeneity in animal studies.

## Inclusion and exclusion criteria

### inclusion

– A normothermic control group*.
– Induction of global ischaemia in the brain of an adult**, living non‐human animal (mammal).
– Investigation of reduction of temperature in relation to global ischaemia

AND

Reporting of:

− Histological outcome assessment of neuronal cell death/injury in brain tissues***

OR

− Neurobehavioural assessment of outcome

OR

– Mortality assessment of the temperature reduction
* Other control groups will be accepted. Please see “Data analysis plan → Quantitative → Stratifications” for a pre‐defined priority list for extraction.
** Animals assumed adult unless manuscript says it is modelling neonatal responses. Age will be recorded if it is given.
*** To avoid bias, an experienced preclinical researcher (TD) will evaluate the validity of staining protocols for determining neuronal cell death/injury blinded to the outcomes. Validity will be assessed based on the choice of stain(s) and the choice of structure(s) evaluated. This will include protocols with appropriate staining for neuronal cell death/injury and appropriate structure and exclude protocols evaluating non‐specific cell distress or glial activity. TD may consult TC if there is uncertainty about the validity of staining.

### exclusion

– Use of historical controls.
– Temperature reduction is induced pharmacologically.
– Cooling/heating was used only to prevent spontaneous temperature change without a corresponding control group.
– Data could not be used for meta‐analysis, for example, no information on group size or variance.
– Studies using animals treated with therapies adjuvant to the temperature reduction will be excluded, but where it is possible to extract data for a temperature reduction group without adjuvant therapy and a control group without adjuvant therapy, these will be included.
– The study researches the benefits of temperature reduction to treat newborns.
– Studies exploring either deep hypothermic circulatory arrest or cardiopulmonary bypass together with temperature reduction are excluded if they do not specifically explore these as treatments of cardiac arrest.

## Search Strategy

Embase & PubMed were searched with no restriction on time of publication or language.

Rationale for search strategy: It was considered unlikely that a study fulfilling the specified inclusion criteria would not contain 1) a synonym to cardiac arrest or ischaemia (broad search), 2) a synonym to hypothermia or temperature reduction (in title or abstract) and 3) a synonym to the brain or its structures (broad search).

Search strategy for PubMed:

(cardiac arrest OR circulatory arrest OR ischemia OR ischaemia OR hypoxia OR anoxia OR infarct OR infarction OR asystole OR resuscitation) **AND** (hypothermia[Title/Abstract] OR hyperthermia[Title/Abstract] OR normothermia[Title/Abstract] OR temperature[Title/Abstract] OR thermoregulatory[Title/Abstract] OR thermoregulation[Title/Abstract] OR chill therapy[Title/Abstract] OR cooling[Title/Abstract] OR cryotherapy[Title/Abstract]) **AND** (brain OR hippocampus OR thalamus OR striatum OR cortex OR neuroprotecti* OR cerebral OR cerebrum OR neuron OR neuronal)

The entire string was pasted into the search field.

Search strategy for Embase:

(’cardiac arrest’ OR ’circulatory arrest’ OR ischemia OR ischaemia OR hypoxia OR anoxia OR infarct OR infarction OR asystole OR resuscitation) **AND** (’hypothermia’:ab,ti OR ’hyperthermia’:ab,ti OR ’normothermia’:ab,ti OR ’temperature’:ab,ti OR ’thermoregulatory’:ab,ti OR ’thermoregulation’:ab,ti OR ’chill therapy’:ab,ti OR ’cooling’:ab,ti OR ’cryotherapy’:ab,ti) **AND** (brain OR hippocampus OR thalamus OR striatum OR cortex OR neuroprotecti* OR cerebral OR cerebrum OR neuron OR neuronal)

The entire string was pasted into the search field with the setting “search as broadly as possible”.

The search strategy was discussed with a librarian. Multiple variations of both synonyms and block constructions were tested before arriving at the final search strategy.

## Data Collection Processes

Articles from the Embase and PubMed searches will be entered into EndNote X7 software. The EndNote library will be exported to CAMARADES software, which will be used for reviewing abstracts and full‐text articles. Duplicate articles will be removed both automatically (using EndNote function “Remove duplicates”, author‐year‐title‐reference type) and manually as the same article may be referenced differently between Embase and PubMed.

Independent reviewers will screen for relevant articles looking at title and abstract; each abstract will be screened by two independent reviewers. HO, GT and HW are primarily responsible for the screening. These lists will be merged and included articles will be reviewed in full text. Full‐text review will be performed independently by two independent reviewers (HO and GT). Articles not meeting all inclusion criteria or fulfilling one or more exclusion criteria will be excluded. Reason for exclusion will be noted. Discrepancies between the three reviewers regarding inclusion/exclusion of full‐text articles will be solved by a further review by one of three additional reviewers (TD, NN or JR), and, if necessary, a consensus discussion will follow. A flow diagram of included and excluded studies at each stage will be presented in accordance with PRISMA guidelines.^20^ A hand search of the reference lists in the included articles will be performed, looking for articles investigating temperature reduction in relation to global ischaemia. As a final complement to the database search, reviews on the topic will be searched for relevant original articles; this will be at the discretion of senior investigators. The database search will be updated towards the end of the project to allow for newly published articles to be included, if applicable.

All included articles will be assessed qualitatively, quantitatively and with a modified STAIR criteria. Data extraction will be performed independently by HO and GT. Disagreements will be solved by discussion, with the help of TD if necessary. Aside from publication ID, author surname and initials, journal and year of publication, qualitative, quantitative and modified STAIR‐criteria data will be extracted in a pre‐defined data extraction sheet (see below). Regarding quantitative data, several stratifications will be recorded (see below). If data are only presented in graphical form, then computerized ruler software will be used to measure graphs (FlexRuler 2.3, DropFrame). If data are incomplete, we will endeavour to contact the authors.

## Data Analysis Plan

### qualitative

Individual studies will be checked using eight criteria outlined below that were derived from multiple sources—the CAMARADES quality checklist,^21^ the checklist for assessing quality in studies researching the effects of hypothermia in focal ischaemia models by van der Worp et al.^22^ and one additional criteria, number five.

1. Publication in a peer‐reviewed journal.
2. Randomization to treatment or control.
3. Blinded induction of ischaemia (i.e. concealment of treatment group allocation at time of induction of ischaemia).
4. Blinded assessment of outcome.
5. Statement of inclusion and exclusion of animals from the study.
6. Sample size calculation.
7. Statement of compliance with regulatory requirements.
8. Statement regarding possible conflicts of interest.

We will count the number of checklist items scored by each publication. If there is any doubt whether a study fulfils a certain criteria, no points will be given. Note that this is not an ordinal score but rather a number of checklist items scored.

### quantitative

We plan to:

- Use a random effects model as we expect great inter‐study heterogeneity.
- Use an I‐square to measure heterogeneity for the overall estimate of each outcome.
- Set a lower limit of 10 publications to perform the meta‐analysis.

Publication bias will be measured with funnel plot, Egger regression and trim and fill.

CAMARADES software will be used for data analysis.

#### Effect sizes

Our primary outcome is neurobehavioural outcome; histological outcome and mortality are secondary. If more than one histological outcome or more than one behavioural outcome is reported from the same cohort of animals, we will summarize these using fixed‐effects meta‐analysis to provide a summary estimate of histological or behavioural outcome for that cohort. In each study, the number of animals per group, mean outcome and standard error or standard deviation for both control and treatment group will be extracted. When a single control group serves multiple treatment groups, the control group will be divided by the number of treatment groups that are included in the meta‐analysis.^23^ If outcome is measured serially (behaviour, histology, mortality), then the final measure will be extracted for meta‐analysis. Ordinal data will be analysed as continuous variables for the purpose of the meta‐analysis.

For neurobehavioural and histological outcome, it is unlikely that we can infer what a “normal” animal would score; therefore, we will use standardized mean difference (SMD) and not normalized mean difference (NMD).^23^ For mortality, we will use odds ratio.

#### Stratifications

The following variables, and their effect on outcome, will be recorded from each study:

The study design will be made up of 16 items: temperature reduction (8 items); ischaemia (2 items); and other (6 items).

Temperature reduction (8 items):

– Timing of temperature reduction (pre‐, intra‐, post‐ischaemia).
– Time to treatment (pre‐treatment, minutes post‐ischaemia).
– Method of inducing temperature reduction (intra‐ or extracorporeal or permissive).
– Depth of temperature reduction (temperature measured closest to brain).
– Duration of temperature reduction (start of temperature reduction defined as start of cooling and end of temperature reduction defined as end of cooling).
– Rate of re‐warming (degrees/hour; start of re‐warming defined as end of cooling and end of re‐warming defined as the point when normothermia is reached or alternatively when re‐warming is actively ended; temperature difference is defined as normothermia minus target temperature).
– Method of re‐warming (intra‐ or extracorporeal or spontaneous).
– Method of controlling temperature in the control group (temperature management—normothermia or hyperthermia or no intervention*).
* See below

Ischaemia (2 items):

– Model of global ischaemia (selective arrest of cerebral circulation, that is 2‐vessel occlusion, 4‐vessel occlusion or cardiac arrest by cardioplegic agents, exsanguination, asphyxia etc.).
– Duration of ischaemia.

Other (6 items):

– Time to outcome assessment (neurobehaviour, histology, mortality).
– Species.
– Strain.
– Sex.
– Comorbid animals (diabetic, aged, hypertensive).
– Choice of anaesthetic (see Appendix 1).

Study quality (1 + 4 = 5 items, quality checklist items numbers 2, 3, 4 + 5):

– Total study quality (according to quality checklist).
– The specific components of the quality checklist.

Stratifications are divided into two domains: study design and quality. These stratifications will be presented in a forest plot with point estimates and 99% (quality) and 99.69% (design) confidence intervals.

We will measure the significance of differences between n groups by partitioning heterogeneity and by using Chi‐square test, n‐1 df. To allow for multiple comparisons, we will set a significance level at p < 0.01 for the quality domain (n = 5) and p < 0.0031 for the design domain (n = 16); the p values were arrived at using the Holm–Bonferroni correction. As we use the statistically more conservative SMD, we will partition heterogeneity for balance instead of using the more conservative meta‐regression.^23^ For continuous variables, we will divide these into quartiles for partitioning of heterogeneity.

If a study uses more than one control (e.g. induced hypothermia vs. induced hyperthermia vs. induced normothermia), we will choose to extract the comparison(s) according to a pre‐defined list of priority:

1. Induced hypothermia versus induced normothermia.
2. Induced hypothermia versus no intervention*.
3. Induced hypothermia versus induced hyperthermia**.
4. Permissive hypothermia versus induced normothermia.
5. Permissive hypothermia versus induced hyperthermia.

* No intervention being either a) animals are allowed to spontaneously remain at normothermia after global ischaemia or b) animals are allowed to spontaneously increase their temperature after global ischaemia or c) animals are allowed to spontaneously decrease their temperature after global ischaemia or d) unknown.

One experiment cannot contribute two different statements about the same intervention (e.g. in the same study, induced 30 °C vs. induced 37 °C would be extracted with greater priority than induced 30 °C vs. induced 40 °C).

** The comparison of induced hypothermia versus induced hyperthermia is included as we anticipate that some studies may use this to mimic the clinical scenario of post‐ischaemic fever. Therefore, we believe that this comparison reflects our primary research question.

### pre‐specified sensitivity analysis

We will perform sensitivity analysis if more than 150 outcomes are reported; where NMD calculation of effect sizes is feasible, we will perform a sensitivity analysis using NMD with meta‐regression to explore the significance of differences between groups of studies. During the review process, we also anticipate identifying issues regarding suitability for sensitivity analysis—due to study variations that we cannot anticipate at the protocol stage.

### modified stair evaluation

We will use modified STAIR criteria to assess the scope of testing in the included studies, thus elucidating the preclinical evidence. This modified STAIR criteria is similar to the one constructed by O'Collins et al. to evaluate neuroprotective drugs in models of focal ischaemia.^24^^,^ 25 We make a few departures from the original STAIR recommendations; we do not demand measures concerning quality control (randomization and blinding as these relate to quality within individual studies, and our objective focuses on a cohort of studies). Some of the items in the study by O'Collins et al. are not applicable to our study, and we also added one item, a global ischaemia model.

**Figure 1:**
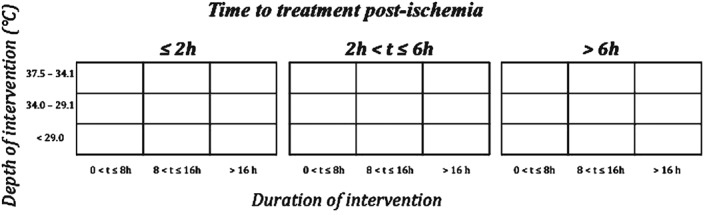
Each box, that is, a combination, can get a modified STAIR score from zero to eight points. If target temperature is presented as XX degrees +/− Y degrees, XX will be used as depth of intervention. If duration of intervention is presented as XX min +/− Y min, XX will be used as duration, and the same applies to time to treatment.

A clinically relevant temperature reduction will always have a specific combination of time to treatment, depth and duration. We will construct three matrixes (<2 hours time to treatment, between 2 and 6 hours, >6 hours), each with three durations and depths. Note that this allows for one study to contribute with more than one combination if it evaluates different depths, durations or timings of temperature reduction. Other combinations of timing (pre‐ and intra‐ischaemic) are possible and will be recorded but not shown in the form of a matrix. An example is given below (see Figure 1). The least temperature reduction from normothermia that we record will be regarded as the upper limit of depth.

The modified STAIR criteria consist of the following:

1. Laboratory setting—Combination tested in two or more laboratories*.
2. Animal species—Combination tested in rodents and gyrencephalic species.
3. Health of animals—Combination used in comorbid animals**.
4. Sex of animals—Combination tested in male and female animals.
5. Outcome measures—Combination evaluated with both histology and behaviour.
6. Long‐term effect—Combination has evaluated long‐term outcome (either histology, mortality or behaviour, 4 weeks or more after ischaemia).
7. Route of delivery—Combination tested with two or more methods of temperature reduction (e.g. intravascular cooling, extracorporeal cooling etc.).
8. Global ischaemia model—Combination tested in two or more models of global ischaemia where at least one model of global ischaemia is accomplished by induced cardiac arrest
  * By different investigators AND in separate laboratories.
  ** Diabetic, aged, hypertensive.

For each publication, we will count the number of checklist items scored. If there is any doubt whether a combination fulfils a certain criterion, no points will be given. Note that this is not an ordinal score but rather a number of checklist items scored.

NB Draft only. Temperature ranges in the three categories may be varied during the full‐text review and data extraction stages, as applicable.

### missing data

#### Qualitative

If an article does not mention a qualitative feature in our data extraction sheet, it will either be presumed not to have been performed (e.g. no mention of randomization will be interpreted as if it was not performed) or considered unknown (e.g. no mention of the sex of the animal used).

#### Quantitative

If an article does not mention a quantitative feature in our data extraction sheet, it will be considered unknown and excluded from that analysis.

## Data Extraction Sheet

NB Draft only. Further extraction fields may be added during the full‐text review and data extraction stages, as applicable.

**Table:** 

Items	Study 1	Study 2	Etc
Publication ID			
Name of principal author/investigator			
Year of publication			
Journal			
*Quality checklist*			
Publication in a peer‐reviewed journal			
Randomization to treatment or control (description)			
Blinded induction of ischaemia (description)			
Blinded assessment of outcome (description)			
Statement of inclusion and exclusion of animals from the study (and number of animals included/excluded and for what reason)			
Sample size calculation			
Statement of compliance with regulatory requirements			
Statement regarding possible conflicts of interest			
*Outcomes*			
Number of animals per group (sham, control and intervention)			
Number of groups			
Mean outcome and standard deviation (histology, neurobehaviour and mortality)			
Method of neurobehavioural evaluation (description)			
Method of histological evaluation (description)			
*Stratifications*			
Timing of temperature management (pre‐, intra‐, post‐ischaemia)			
Time to treatment (post‐ischaemia)			
Method of inducing temperature reduction (intra‐ or extracorporeal or permissive)			
Depth of temperature reduction (temperature measured closest to brain)			
Duration of temperature reduction (start of temperature reduction defined as start of cooling and end of temperature reduction defined as end of cooling)			
Rate of re‐warming (degrees/hour; start defined as end of cooling and end of re‐warming defined as the point when normothermia is reached or alternatively when re‐warming is actively ended; temperature difference is defined as normothermia minus target temperature)			
Method of re‐warming (intra‐ or extracorporeal or spontaneous)			
Method of controlling temperature in the control group (temperature management—normothermia or hyperthermia or no intervention*)			
			
Model of global ischaemia (selective arrest of cerebral circulation, e.g. 2‐vessel occlusion, 4‐vessel occlusion or cardiac arrest by cardioplegic agents, exsanguination, asphyxia etc.)			
Duration of ischaemia			
Time to outcome assessment (neurobehaviour, histology, mortality)			
Species			
Strain			
Sex			
Comorbidities (diabetic, aged, hypertensive)			
Choice of anaesthetic			
*Modified STAIR evaluation*			
Laboratory setting (which laboratory/investigator was primarily responsible for the study)			
*Intervention–Control set up* (e.g. induced hypothermia vs. induced normothermia etc.)			
Comparisons of intervention–control in experiment			

**Contribution of Authors**

HO—Hilmer Olai

GT—Gustav Thornéus

HW—Hannah Watson

MM—Malcolm Macleod

HF—Hans Friberg

JR—Jonathan Rhodes

NN—Niklas Nielsen

TC—Tobias Cronberg

TD—Tomas Deierborg

The idea for the project was developed by TD, TC, NN and HF. This protocol was written in the spring and summer of 2015 by HO and GT, with input from HF, NN, TC, TD, MM, JR and HW.

Screening of abstracts (independently)—primarily HO, GT and HW.

Full‐text review (independently)—HO and GT.

- Disagreements regarding inclusion/exclusion—TD, NN and JR.
- Evaluation of histological protocols (blinded to outcome)—TD with support from TC.
- Hand search of reference lists in included articles and reviews (independently)—HO, GT and HW.

Data extraction (independently)—HO and GT

Data analysis—Junior and senior investigators

Write report—Junior and senior investigators

## Conflict of Interest

The authors have declared no conflicts of interest for this article.

## STRATIFICATIONS → STUDY DESIGN → CHOICE OF ANAESTHETIC

**Table:**

		Study 1	Study 2	Etc.
Time of Administration**	Before			
	During			
	After			
	Unknown*			

* Unknown time of administration.

** We will make a note “W.O.” if the drug is washed out.
